# Processing implicit control: evidence from reading times

**DOI:** 10.3389/fpsyg.2015.01629

**Published:** 2015-10-27

**Authors:** Michael McCourt, Jeffrey J. Green, Ellen Lau, Alexander Williams

**Affiliations:** ^1^Department of Philosophy, University of MarylandCollege Park, MD, USA; ^2^Department of Linguistics, University of MarylandCollege Park, MD, USA

**Keywords:** anaphora, implicit control, implicit argument, rationale clause, self-paced reading

## Abstract

Sentences such as “The ship was sunk to collect the insurance” exhibit an unusual form of anaphora, implicit control, where neither anaphor nor antecedent is audible. The non-finite reason clause has an understood subject, PRO, that is anaphoric; here it may be understood as naming the agent of the event of the host clause. Yet since the host is a short passive, this agent is realized by no audible dependent. The putative antecedent to PRO is therefore implicit, which it normally cannot be. What sorts of representations subserve the comprehension of this dependency? Here we present four self-paced reading time studies directed at this question. Previous work showed no processing cost for implicit vs. explicit control, and took this to support the view that PRO is linked syntactically to a silent argument in the passive. We challenge this conclusion by reporting that we also find no processing cost for remote implicit control, as in: “The ship was sunk. The reason was to collect the insurance.” Here the dependency crosses two independent sentences, and so cannot, we argue, be mediated by syntax. Our Experiments 1–4 examined the processing of both implicit (short passive) and explicit (active or long passive) control in both local and remote configurations. Experiments 3 and 4 added either “3 days ago” or “just in order” to the local conditions, to control for the distance between the passive and infinitival verbs, and for the predictability of the reason clause, respectively. We replicate the finding that implicit control does not impose an additional processing cost. But critically we show that remote control does not impose a processing cost either. Reading times at the reason clause were never slower when control was remote. In fact they were always faster. Thus, efficient processing of local implicit control cannot show that implicit control is mediated by syntax; nor, in turn, that there is a silent but grammatically active argument in passives.

## Background

Sometimes an aspect of speaker meaning has unclear provenance. Is it semantic or pragmatic? Is it or is it not determined, that is, by the structural identity of the sentence itself? In such cases online measures may help us find the source of the meaning, as the two routes to interpretation may take measurably different paths.

One familiar example comes from verb phrase ellipsis, as in (2). After (1), the speaker of (2) means that the Yankees traded an outfielder. But is this decided by the structural identity of his sentence token?

(1) The Red Sox traded an outfielder.
(2) The Yankees did too.

Many answer *yes* (Sag, 1976; Williams, 1977; Fiengo and May, 1994; Merchant, 2001). They say that this use of (2), unlike others, has the verb phrase *trade an outfielder*, with all the structure of the verb phrase in (1), just silent. Others answer *no* (Dalrymple et al., 1991; Hardt, 1993; Ginzburg and Sag, 2000; Culicover and Jackendoff, 2005). Every use of (2), they say, has an unstructured verb phrase that simply means *P*, where *P* is a free variable over properties. The value of that variable is then decided “by context,” not by the sentence itself. On the first account, the string in (2) is ambiguous between infinitely many sentences, each with a different verb phrase and hence a different meaning. On the second, it has a single meaning that is sensitive to context. These two routes to interpretation—semantic vs. pragmatic, disambiguation vs. anaphora, recovery of structure vs. resolution of a variable—might involve different cognitive processes, and might also register differently in some online processing measure. If they do, that measure may provide some evidence for which account is correct, a question that remains contentious. Accordingly, a rich body of literature has pursued this idea (Tanenhaus and Carlson, 1990; Shapiro and Hestvik, 1995; Frazier and Clifton, 2001, 2005; Martin and McElree, 2008; Kertz, 2010; Yoshida et al., 2012; see Phillips and Parker, 2014 for an overview).

We explore another area in this same light, namely *implicit control of reason clauses*, on display when we use (3) to mean (5).

(3) The candidates were interviewed to find the best person for the job.
(4) Someone interviewed the candidates in order to find the best person for the job.
(5) Someone_k_ interviewed the candidates in order for them_k_ to find the best person for the job.

Both (3) and (4) have an infinitival *reason clause* with the verb *find*, adjoined to a *target clause* with the verb *interview*. A reason clause, or *rationale* clause (Faraci, 1974; Jones, 1985), offers a teleological explanation of the fact expressed by its target clause. Why were the candidates interviewed, according to this use of (3)? Because then the interviewers might find the best person for the job. The understood subject of a reason clause, called PRO, may be construed anaphorically, as denoting a thing previously mentioned or implied. Anaphora involving PRO is called *control*, though we commit to no analysis with this term. When (3) is used to mean (5), PRO names the interviewer entailed by the verb in the target clause, *interview*. But the interviewer is named by no audible dependent in that clause; (3) is a *short* passive, with no *by*-phrase. So here control is *implicit*. Control is *explicit* when we use (4) to mean (5). Now the interviewer is audibly realized, here as the subject of an active target clause.

On the *standard theory* of implicit control (Roeper, 1987), the relation is not pragmatic, but syntactic and therefore semantic. Specifically, it is encoded in the context-invariant meaning of the two-part sentence that combines the reason clause and its target clause host; and this encoding goes by way of a syntactic dependency, *binding*^1^, which effects sameness of reference. Binding links PRO in the reason clause to a postulated silent argument in the passive target clause, providing PRO with an antecedent.

Semantically, the silent argument is linked to the *deep-S role* of the verb: the semantic relation assigned to the subject of an active clause with that verb. For *interview*, this is the role of interviewer. Syntactically, the silent argument has one of two representations, depending on the analysis of the passive. It may be a formal feature of the verb, part of a feature array that syntactically indexes certain semantic properties, perhaps a “Theta Grid” (Stowell, 1981), “Argument Structure” (Grimshaw, 1990; Manning and Sag, 1998), or “Logical Structure” (van Valin, 1990). Or it may be a separate expression that combines with the verb in syntax (Baker et al., 1989; Stanley, 2000). Either way, the silent argument serves here to provide PRO with a formal antecedent. This allows PRO to be bound, and hence for implicit control to be fixed syntactically, and thus in the compositional semantics. In this way implicit control is assimilated to the paradigm cases of control, where PRO must be coreferent with a particular argument in the next clause up. In (6) or (7), for example, it is must be coreferent with the subject of the *promise* or *rob* clauses, respectively.

(6) Lee heard Mo promise PRO to leave.
(7) Lee robbed Mo while PRO distracting her.

This theory has a good motive. Many restrictions on control of reason clauses, or *reason control*, can be described in syntactic terms (Keyser and Roeper, 1984; Roeper, 1987). When reason control is explicit, the antecedent can be the subject but not the object of its clause (Williams, 1974)^2^. Thus, we can use (8) but not (9) to talk about how the sharks have their gills kept clean, since *these sharks* is the subject in (8) but the object in (9). Conversely only (9) implies that the parasites have gills.

(8) These sharks cover themselves with parasites to have their gills kept clean.
(9) Parasites cover these sharks to have their gills kept clean.

The antecedent can also be a *by*-phrase, when the target clause is a long passive. Thus, we can use (10) to convey that the Red Sox hoped to acquire a better pitcher in trading two outfielders.

(10) Two outfielders were traded by the Red Sox to acquire a better pitcher.

But the right conclusion is not that the antecedent must be assigned the deep-S role of the verb in the target clause. This is not necessary (Williams, 1974; Zubizaretta, 1982; Roeper, 1987), as shown by (11), which can be used to mean that Lisa was arrested so that *she* might seem like a radical.

(11) Lisa was arrested just to seem like a radical.

The better conclusion is that explicit control must be by a *subject*, so long as we presume that a *by*-phrase counts as a subject for at least these purposes. Let us use the term *S* for an argument that “counts as a subject” in this sense, so that reason control must be by an S when explicit. Then we can describe implicit control in analogous terms, if we link the deep-S role in a short passive to a silent S argument, called “implicit” because it is grammatically active. This is the standard theory.

Standard theory in hand, we have a syntactic account of some cases where implicit control is impossible. Sentence (12) describes the theft of a ship, and therefore entails a victim from whom the ship was stolen. But we cannot use (12), it seems, to say that the entailed victim was the intended collector of the insurance, even if he hired the crook for this very purpose. On the standard theory, this is because the role of victim is not linked to an implicit S. And this conclusion is well-justified, since the victim role is assigned to the subject in neither actives nor passives with *steal*.

(12) A hired crook stole the ship to collect the insurance.

Middles, such as (13a), receive a more stipulative account. In a middle as in a short passive, the deep-S role of the verb is assigned to no audible dependent; no audible part of (13a) refers to killers, for example. But with middles this role can never antecede a reason clause PRO (Keyser and Roeper, 1984; Roeper, 1987; Mauner and Koenig, 2000). After (13a), for example, we cannot use (13b) to say that the winter survival of the *killers* explains why prey animals kill easily in the autumn.

(13a) In the autumn the prey animals kill easily.
(13b) #to survive the winter without hunger.

To capture this, the standard theory stipulates a difference in argument structure. In a middle, it says, the deep-S role is not linked to an S, unlike in a passive.

These conclusions have broader implications beyond the analysis of reason clauses, as they make it more plausible that an argument may be silent but grammatically active (Stanley, 2002)^3^. But the standard theory leaves several questions unanswered. It suggests no reason why the implicit S in a passive does not always function as a subject, in relation to all types of adjunct clauses (Vinet, 1988; Iwata, 1999; Landau, 2000), not just reason clauses. By hypothesis (14) has a silent S in the role of thief, and yet we cannot use (14) to mean that my wallet was stolen while the *thief* was distracting me, letting this implicit S control the non-finite temporal adjunct.

(14) My wallet was stolen while distracting me.

The standard theory is also silent on why implicit control is not available to the deep-S role of every passive clause. The meaning that is unavailable to (14) is also unavailable to (13) (Williams, 2015). Yet (15a) is a passive, not a middle, and so should have an implicit S in the role of killer.

(15a) In the autumn the prey animals are killed easily
(15b) #to survive the winter without hunger.

Nor can the standard theory accommodate data like (16) (Williams, 1985, 2015; Lasnik, 1988). Sentence (16) can be used to convey that a young girl cut the ribbon so that the organizers of the event might acquire the support of female voters (Williams, 2015). Yet in a clause with *cut*, there is no argument that stands for organizers of the cutting, as distinct from the cutters.

(16) A young girl cut the ribbon just to acquire the support of female voters.

Finally, the standard theory cannot account for what we call *remote control*, to which we turn in a moment.

Given these doubts, we should welcome additional evidence for the standard theory; and some has been offered in the previous psycholinguistic literature. In a series of stop-making-sense and self-paced reading time studies, Mauner et al. (1995) compared implicit with explicit control of reason clauses. They did so by comparing reason clauses following active, full passive, short passive and intransitive target clauses (15–18).

(17) Someone sank the ship to collect the insurance.
(18) The ship was sunk by someone to collect the insurance.
(19) The ship was sunk to collect the insurance.
(20) The ship sank to collect the insurance.

No differences in acceptability judgments or in reading times were observed in the reason clause in conditions (15–17), but significantly slower reading times and more “unacceptable” responses were observed following the intransitive (20). Mauner and colleagues took these results to support the standard theory of implicit control, on the basis of the following reasoning. First, something like the standard theory of explicit control was assumed: in active examples like (17), PRO is locally bound by the surface subject of the target clause. It was then assumed that finding similar processing profiles for two interpretive dependencies—such as implicit vs. explicit control—would provide evidence that the same mechanisms are at work in resolving them both. Since explicit control by the surface subject of an active target clause is supposed to be mediated syntactically, and since no behavioral differences were observed between explicit (15, 16) and implicit control conditions (19), these earlier results were taken to support the standard view that implicit control is syntactic binding of PRO by a silent argument in the short passive.

Although Mauner and colleagues' results have been taken to constitute important evidence in favor of the standard view of implicit control, this interpretation relies on the assumptions outlined above. In the current study, we test these assumptions further by examining the case of *remote* control. Prior studies considered only *local* control, where the target and reason clauses are syntactically dependent, forming a single sentence. In *remote* control (Higgins, 1973; Sag and Pollard, 1991; Williams, 2015), as in (21), the two clauses are independent, in two separate sentences. But we can still use (21) to mean (5).

(21) The candidates were interviewed. The goal was to find the best person for the job.
(5) Someone_*k*_ interviewed the candidates in order for them_*k*_ to find the best person for the job.

In remote control, the infinitival clause is the complement to an equative (or specificational) copula, in a sentence that is separate from the target clause. The subject of the target clause is something like *the goal, the reason*, or *the purpose*, a description with a relational noun. We understand that, here, this description is used to refer to a relation that is directed at the target fact, taking *the goal* in (21), for example, to refer to the goal of interviewing the candidates.

Crucially, remote control shows exactly the same restrictions as local control (Williams, 2015). Among others, the contrasts in (1–13) are all preserved when control is remote. (8′) and (9′) show that subjects, but not objects, can be implicit controllers in remote configurations; only (9′) implies that parasites have gills.

(8′) These sharks cover themselves in parasites. The goal is to have their gills kept clean.
(9′) Parasites cover these sharks. The goal is to have their gills kept clean.

And, as with (12) above, it is not possible to use (12′) to mean that the hired crook stole the ship so that his employers could collect the payout.

(12′) A hired crook stole the ship. The reason was to collect the insurance.

Yet here these patterns cannot be explained in terms of syntactic binding. Binding cannot cross independent sentences, and the reason clause, when remote, is syntactically separate from its target. Conceivably—though we do not think that this is correct, for reasons we discuss elsewhere (Green and Williams, in preparation)—the copular clause has hidden structure that conceals a local (same-sentence) binder for PRO, one that is itself anaphoric to an S in the target clause^4^. But even if it did, the anaphoric relation between this local binder and its antecedent in the target clause would still be intersentential. Hence, whatever it is that underlies the interpretive dependency between PRO and the implied interviewer in (21), it cannot be syntactic binding.

The anaphora in (21) must therefore be pragmatic. PRO in a remote reason clause—or, on the alternative that we reject, its hidden local binder—must function not like a bound pronoun, but like a free pronoun or definite description. In turn, the limits on its interpretation cannot be explained directly in terms of structure in the target clause. Rather, its domain of reference must be highly restricted, in terms that only correlate, partially and indirectly, with subjecthood in the target clause^5^. Examples such as (12) suggest that a notion of *responsibility* may be relevant: perhaps PRO in a reason clause, as a matter of the meaning of the construction, ranges only over parties viewed as explanatorily *responsible* for the fact it is meant to explain, a class that may but need not include the individual in the deep-S role to the event of the verb (often, its agent)^6^. However, the grammatical analysis of remote control is beyond the scope of the current work. Here, the key observation is just that the restrictions on local and remote control appear to be identical. Since remote control must be pragmatically mediated, this weakens the motive for a semantic, hence syntactic, account of local control, and at the same time provides a new means of examining the extent to which reading time measures provide support for the syntactic account. If the standard theory is correct, then different mechanisms must be at work in resolution of local (one-sentence) and remote (two-sentence) control: syntactic binding and something like free pronoun interpretation, respectively. On the other hand, if what we now call the *pragmatic theory* is correct, then something like free pronoun interpretation supports resolution not only of remote control, but also of local control.

In the current study, we investigate these alternative hypotheses by examining processing measures in a series of self-paced reading time studies comparing remote and local reason clauses, with and without explicit antecedents. The predictions are the following. Since the standard theory proposes different mechanisms for resolving local and remote control, and the pragmatic theory proposes the same mechanism, the standard theory predicts differences in the processing of local and remote control, while the pragmatic theory does not. These differences might be realized in several ways. First, following the logic in Mauner et al. (1995), implicitness may be costly in forming pragmatic dependencies (because a referent must be inferred), but not costly in forming syntactic dependencies (because binding to the syntactic argument position proceeds in exactly the same way whether it is audible or not). Given this assumption, the standard theory would predict in the current experiments an interaction between implicitness and distance: an effect of implicitness should be present in the pragmatically mediated remote conditions but not in the syntactically mediated local conditions. Second, pragmatically mediated or syntactically mediated dependencies are likely to differ in processing cost independent of implicitness because they involve reference to different kinds of memory representations. Therefore, in the current experiments, the standard theory could also predict a main effect of distance, but the direction of this difference depends on the linking hypothesis assumed. If syntactic binding is more costly to resolve than free pronoun interpretation, then the effect of implicitness should be larger in remote control. If free pronoun interpretation is more costly, then the effect of implicitness should be larger in local control. As previous psycholinguistic work does not provide clear predictions about which should be more costly (see Frazier and Clifton, 2000, for discussion), either could be taken to be consistent with the standard theory, although it could also be the case that binding and free pronoun interpretation do not differ fundamentally in processing cost (Cunnings et al., 2014).

The pragmatic theory does not predict any difference in the costs of implicitness in remote vs. local control. If we observed no such differences, this could be due to the fact that local and remote control are both mediated by the same kind of pragmatic mechanism. However, such a conclusion would be too strong here. It might be that reading times in particular are not a sensitive enough measure to detect differences between local and remote control that other measures might detect. Or it could be that processing cost is more generally not a reliable diagnostic of whether a dependency is semantically or pragmatically mediated. However, it is important to remember that Mauner et al.'s (1995) finding of no processing cost for local implicit vs. explicit control is one of the key pieces of evidence currently taken to support the standard theory, and that reading time was one of the online measures used in that study. Skepticism about the ability of self-paced reading to detect differences in processing of local and remote control would thus undermine earlier arguments in favor of the standard theory. These relied on the premise that, in fact, behavioral measures could reflect differences in processing as a function of whether a dependency was semantically or pragmatically mediated. Thus, if we observe no differences in processing of local vs. remote reason clauses, we can at the very least conclude that these earlier results do not in fact provide evidence for the standard account.

## Experiments

### Experiment 1

The goal of Experiment 1 was to test whether differences obtain in processing of local vs. remote control, or between implicit and explicit control within local vs. remote configurations. Experiment 1 manipulated explicitness with passive sentences that varied in the presence or absence of a *by*-phrase that explicitly named the agent of the event described by the passive. Observation of differences in processing of remote and local control, or between implicit and explicit control in local vs. remote configurations, would provide support for the standard theory. Should we observe no such differences, this would either raise a challenge for the standard theory, or undermine previous arguments in its favor, as discussed above.

#### Methods and materials

##### Participants

Participants were 38 native speakers of English from the University of Maryland community. Participants gave informed consent, and received credit in an introductory linguistics course or were compensated $5 for their participation in the experiment. All participants were naïve to the purpose of the experiment. The self-paced reading task lasted for approximately 30 min.

##### Materials

Sentences were created by combining a finite passive clause with a non-finite reason clause. In a 2 × 2 design, stimuli varied in whether the target clause contained an overt antecedent for the understood subject of the reason clause (*explicitness*), and in whether the target and reason clauses were syntactically independent (*distance*). In conditions labeled *implicit*, the target clause was a short passive and therefore lacked an overt antecedent for PRO. In conditions labeled *explicit*, the target clause was a passive with a *by*-phrase describing the agent of the event, which served as the antecedent of PRO. The dependency was *local* when the reason clause was syntactically an adjunct of the target clause. The dependency was *remote* when the two clauses were syntactically independent, the reason clause being hosted by a copular clause in a separate sentence. An example set of materials is provided in Table 1.

**Table 1:** **Experiment 1 materials**.

**Regions of analysis**												
	**Pre-target**	**1**	**2**	**3**	**4**	**5**	**6**	**7**	**8**	**9**	**10**	**Post-target**
ex. loc:	The candidates were interviewed	by	the	committee				to	find	the	best	person for the job.
im. loc:	The candidates were interviewed	three	weeks	ago				to	find	the	best	person for the job.
ex. rem:	The candidates were interviewed	by	the	committee.	The	reason	was	to	find	the	best	person for the job.
im. rem:	The candidates were interviewed	three	weeks	ago.	The	reason	was	to	find	the	best	person for the job.

In order to control the position of the reason clause across explicit and implicit conditions, we substituted a temporal adjunct, such as *for several hours*, in the implicit conditions in place of the *by*-phrase. Our materials were also crafted to strongly favor interpreting PRO as the satisfier(s) of the deep-S role in the target clause. To this end, we controlled several properties across item sets. First, the reason clause always expressed a property that can be satisfied by people, but not by facts or events. While people can find the best employees for a job, for example, facts or events cannot. This eliminated the possibility, otherwise readily available (Williams, 1985; Lasnik, 1988), of resolving PRO to the fact or event named by the target clause itself. This can happen in (22), which can be used to say that the candidates were interviewed because interviewing them might make a good impression.

(22) Candidates were interviewed to make a good impression.

Second, our passive target clauses mostly had subjects that were semantically implausible as subjects for the reason clause, lowering the chance that they would be taken to antecede PRO. Third, in general our passive target clauses also resisted being read as “adjectival passives,” as in *the shoes are polished*. This matters, since adjectival passives do not readily support implicit control by the deep-S role of their verb root. And finally, our target clauses never contained first-person, second-person or impersonal pronouns. This lowered the likelihood, however small, that PRO was read as logophoric or impersonal, like English impersonal *one*, denoting a group that shares the interlocutors' perspective.

Twenty-four sets of four items in these conditions were distributed across four lists in a Latin square design. 96 filler sentences were also included, such that each participant read a total of 120 sentences. Approximately half of the fillers were one-sentence fillers. The other half were two-sentence fillers, roughly matching the 1:1 ratio of one- to two-sentence items in the main experimental stimuli. Some of the fillers involved adjectival passives and prepositional *to* in order to reduce the likelihood of within-task effects. Such constructions are syntactically very similar to our experimental items, but are differentiated semantically. Their inclusion was intended as a distraction, to make it less likely that readers would gain familiarity during the task with handling reason clauses.

Each sentence was followed by a comprehension question. Comprehension questions varied in whether they targeted information in the target clause, in the reason clause, or concerning the relation between target and reason clause. This reduced the likelihood of participants developing superficial reading strategies during the task.

##### Procedure

Sentences were displayed on a desktop PC in a moving-window self-paced reading display using the Linger software package (Doug Rohde, MIT). Each sentence initially appeared on a black screen masked by white dashes, with spacing and punctuation intact. Participants revealed the first word by pressing the space-bar on a keyboard. Subsequent words appeared in place of their respective dashes non-cumulatively as participants pressed the space-bar. The order of presentation of target and filler items was randomized for each participant. Participants were instructed to read the sentences carefully and for understanding but at their normal pace. Before the beginning of the experiment, participants were able to gain familiarity with the task with four practice items. Each sentence was followed by a yes/no comprehension question. Incorrect answers to comprehension questions elicited onscreen feedback. The entire procedure took approximately 30 min.

##### Data analysis

The minimum comprehension question accuracy required for inclusion of a participant's data in the analysis was 80%. Data from two participants were excluded due to comprehension question inaccuracy, resulting in a final dataset of 36 participants.

Statistical analysis was performed in regions 1–10, where region 1 was the first region in which conditions differed (the region beginning the *by*-phrase or temporal adjunct prior to the reason clause), and region 7 was the *to* region that began the critical reason clause. We used mixed-effects linear regressions to assess the reliability of the effects associated with the experimental factors. The effects of *explicitness* and *distance* on reading times were tested using linear mixed effects models in R (Bates et al., 2014; R Core Team, 2014), and *p*-values were obtained using lmerTest (Kuznetsova et al., 2014), which uses Satterwaithe approximations to calculate degrees of freedom. Note that regions 4–6 (*The reason was*) were only included in the remote conditions, and therefore tests in these regions necessarily examined only the effect of explicitness.

Reading times above 2000 ms were excluded. This resulted in loss of 0.16% of the data. Reading times were then converted to a log scale for statistical analysis. The fixed effects in the model were the factors *explicitness* (explicit vs. implicit), *distance* (local vs. remote), and their interaction. In addition to these fixed-effects, participants, and items were crossed, starting with random intercepts and slopes, and removing one level of complexity until the model converged with correlations of less than 0.9 in random effects in all regions and experiments described here, following the recommendations of Baayen et al. (2008) and Barr et al. (2013). This resulted in a model including random intercepts for subjects and items, but no random slopes.

#### Results

Mean comprehension question accuracy for experimental stimuli across participants and items was 92% (range across conditions: 91–93%), suggesting that participants were successful in comprehending the main experimental stimuli.

**Figure 1:**
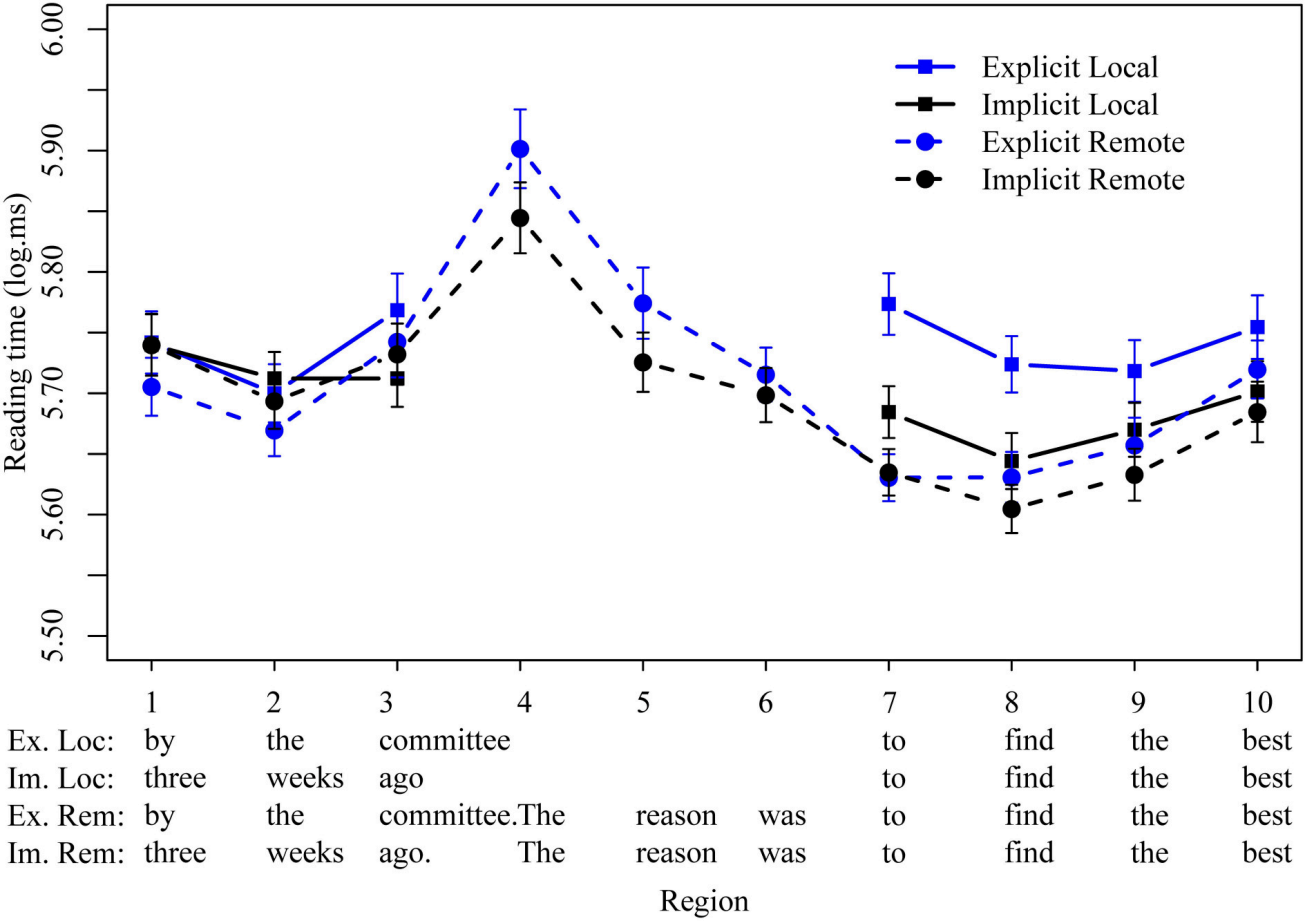
**Mean logged reading times (ms) for all four conditions in regions of interest in Experiment 1**.

Logged reading times are plotted in Figure 1, with significant effects summarized in Table 2. Unexpectedly, in self-paced reading times, the most prominent effect we observe was a slowdown for the explicit local condition relative to the other four conditions. Results of the linear mixed effects models are presented in Table 2. In regions 1–3, immediately following the short passive, we found no significant main effects of either distance (whether the reason clause is local or remote) or explicitness (whether the target clause is a short or long passive) and no interactions, although the explicit local condition was already numerically slowest by region 3. There was also no significant difference between the remote explicit and implicit conditions in regions 4–6 (*The reason was*), although reading times for the explicit condition were numerically longer. However, at the infinitival *to*, region (9), we observed significant main effects of explicitness (β = −0.14, *t* = −6.0, *p* < 0.001) and distance (β = −0.09, *t* = −3.7, *p* < 0.001) and a significant interaction between explicitness and distance (β = 0.09, *t* = 2.8, *p* < 0.01). Follow-up pairwise comparisons indicated that this interaction was driven by a significant effect of explicitness in local conditions (β = −0.09, *t* = −3.3, *p* < 0.001) but not in remote conditions (β = 0.004, *t* = 0.2, *p* > 0.2). In short, there appears to be a strong slowing effect of the *by*-phrase on reading times at the infinitival in the reason clause, but only in one-sentence conditions. In region 8 we observed the same pattern numerically, but here the interaction did not reach significance (main effect of distance: β = -0.09, *t* = -3.9, *p* < 0.001; main effect of explicitness: β = −0.08, *t* = −3.3, *p* < 0.001; interaction between explicitness and distance: β = 0.05, *t* = 1.6, *p* = 0.1). These differences diminished following the main verb in regions 9 and 10, although we observed a main effect of distance in region 9 (β = −0.06, *t* = −2.4, *p* < 0.05) and a marginal main effect of explicitness in region 10 (β = 0.02, *t* = 0.46, *p* > 0.2).

**Table 2:** **Experiment 1 results**.

**Region**	**Factors**	**Estimate**	**S.E**.	***t*-value**	***p*-value**
	Explicitness	−0.089	0.024	−3.73	<0.001
7	Distance	−0.143	0.024	−5.99	<0.001
To	Explicitness × Distance	0.094	0.034	2.77	0.006
	Explicitness	−0.08	0.024	−3.31	<0.001
8	Distance	−0.093	0.024	−3.39	<0.001
Find	Explicitness × Distance	0.054	0.034	1.57	0.116
	Explicitness	−0.048	0.025	−1.92	0.055
9	Distance	−0.061	0.025	−2.43	0.015
the	Explicitness × Distance	0.024	0.036	0.68	0.498

#### Discussion

The goal of Experiment 1 was to investigate differences in the cost of resolving implicit vs. explicit control of reason clauses, in local and remote configurations. In this experiment we used a comparison between short and long passives to manipulate whether the antecedent to PRO was implicit or explicit, respectively. The main effect we observed is unexpected according to either the standard theory or the pragmatic theory: the local explicit condition was slower at the beginning of the reason clause than the other three conditions. The logic in Mauner et al. (1995) would predict the longest reading times for the remote implicit condition, which requires the costly operation of inferring an antecedent for PRO. A more generic version of the standard theory would predict only a main effect of distance. What, then, can explain our results? Why were the longest reading times observed when the antecedent was both explicit, and closer to the position at which the dependency is resolved?

Because this pattern contradicts the predictions of all theories about implicit arguments that we are aware of, we conclude that the slowdown for the explicit local condition is most likely due to an independent factor. In particular, we suggest that this slowdown is an index of continued processing difficulty elicited by the preceding *by*-phrase. Normally *by*-phrases carry narrow focus. That is, we would normally read our long passive example—*The candidates were interviewed by the committee—*with prosodic prominence on *committee*, contrasting the committee with other possible interviewers that might be presently relevant. It has been observed that linguistically focused items elicit slower reading times (Lowder and Gordon, 2015). Furthermore, this effect may be particularly pronounced in the current experiment, because the interpretation of the reason-clause is sensitive to focus (Dretske, 1972): the reason why the candidates were *interviewed*, for example, may not be the reason why they were interviewed *by the committee*. As for why the effect of the *by*-phrase does not obtain in the remote conditions, this is plausibly explained by the availability of more time for processing the focused *by*-phrase prior to the focus-sensitive reason clause during the intervening *The reason was* segment in the remote conditions. Indeed, reading times for the explicit condition were numerically larger during this region.

If this is the correct explanation for the unexpected effects we observe in Experiment 1, then long passives are not ideal as a baseline condition of explicit control, if we want to isolate the specific costs of implicit control. Therefore, in our subsequent experiments, we instead use active transitive clauses for this purpose.

### Experiment 2

Experiment 1 tested the effect of explicitness in local vs. remote control configurations by comparing short and long passives, but we found that the *by*-phrases in long passives introduce independent reading time costs. Experiment 2 therefore manipulated explicitness by comparing short passives with active transitive clauses instead.

#### Methods and materials

##### Participants

Participants were 36 native speakers of English from the University of Maryland community. Participants gave informed consent, and received credit in an introductory linguistics course or were compensated $5 for their participation in the experiment. All participants were naïve to the purpose of the experiment. The self-paced reading task lasted for approximately 30 min.

##### Materials

Twenty-four sets of four target sentences again varied in a 2 × 2 design with the factors *explicitness* and *distance*. However, explicitness is now manipulated by comparing control by short passives with control by active transitive clauses, as shown in Table 3. The same fillers and comprehension questions were used as in the earlier experiments. An example set is provided in Table 3.

**Table 3:** **Experiment 2 materials**.

**Regions of analysis**											
	**Pre-target**	**1**	**2**	**3**	**4**	**5**	**6**	**7**	**8**	**9**	**Post-target**
ex. loc:	The committee interviewed	the	candidates				to	find	the	best	person for the job.
im. loc:	The candidates	were	interviewed				to	find	the	best	person for the job.
ex. rem:	The committee interviewed	the	candidates.	The	reason	was	to	find	the	best	person for the job.
im. rem:	The candidates	were	interviewed.	The	reason	was	to	find	the	best	person for the job.

##### Procedure

The procedure for Experiment 2 was the same as the procedure for Experiment 1.

##### Data analysis

Comprehension question accuracy was above 80% for all participants. Statistical analysis was performed in regions 1–9, where region 1 was two regions prior to the critical word *to* in the local conditions and region 6 was the *to* region that began the critical reason clause. Analysis procedures were the same as described for Experiment 1. The exclusion of reading times above 2000 ms resulted in a loss of 0.2% of the total data. Note that regions 3–5 (*The reason was*) were only included in the remote conditions, and therefore tests in these regions necessarily examined only the effect of explicitness.

#### Results

The mean comprehension question accuracy for experimental items across participants and items was 96% (range across conditions: 95–97%), suggesting that participants were successful in comprehending the main experimental stimuli.

**Figure 2:**
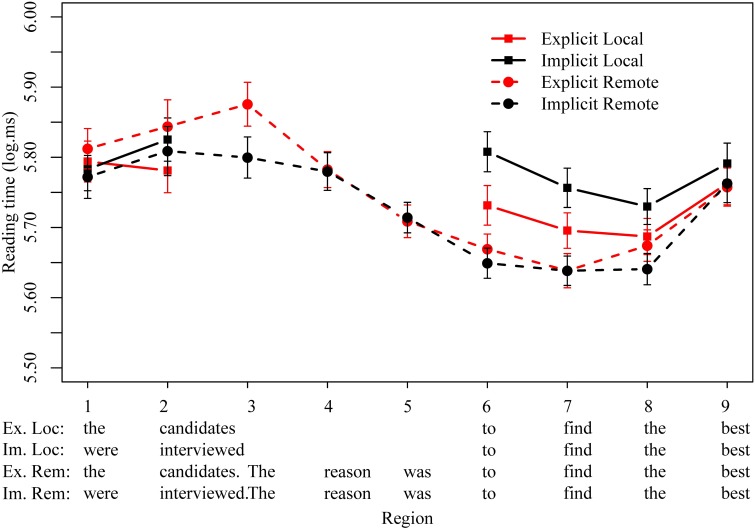
**Mean logged reading times (ms) for all four conditions in regions of interest in Experiment 2**.

Logged reading times are plotted in Figure 2, with significant effects summarized in Table 4. In the regions preceding the reason clause (1–5), the only significant effect observed was slower reading times for the explicit remote condition than the implicit remote condition for the first word of the second sentence, *The* (β = 0.08, *t* = 2.1, *p* < 0.05). Although the reason for this difference is not clear, we note that the conditions come back together in the next region and are very tightly matched prior to the beginning of the critical reason clause.

**Table 4:** **Experiment 2 results**.

**Region**	**Factors**	**Estimate**	**S.E**.	***t*-value**	***p*-value**
	Explicitness	−0.02	0.028	−0.71	0.477
6	Distance	0.062	0.028	2.21	0.027
to	Explicitness × Distance	0.096	0.04	2.42	0.016
	Explicitness	−0.0001	0.027	−0.004	0.997
7	Distance	0.057	0.027	2.13	0.033
find	Explicitness × Distance	0.065	0.038	1.71	0.088
	Explicitness	−0.033	0.025	−1.32	0.186
8	Distance	0.013	0.025	0.52	0.607
the	Explicitness × Distance	0.076	0.036	2.13	0.034

At the infinitival to (region 6), a main effect of distance was observed, with slower reading times for local conditions (β = 0.06, *t* = 2.2, *p* < 0.05), and we also observed an interaction of explicitness and distance (β = 0.09, *t* = 2.4, *p* < 0.05). However, this interaction was not in the direction predicted by the standard theory; rather than implicitness requiring costly inference in the pragmatically-mediated remote condition, we observed a cost of implicitness in the local conditions. Pairwise comparisons show that the implicit local condition was significantly slower than the explicit local condition (β = 0.08, *t* = 2.4, *p* < 0.05), but no differences in reading times were observed for implicit and explicit remote conditions (*p* > 0.2). A similar pattern was observed at the subsequent verb (region 7), with a main effect of distance (β = 0.06, *t* = 2.1, *p* < 0.05) and a marginal interaction between explicitness and distance (β = 0.06, *t* = 1.7, *p* = 0.09). At the region following the verb (region 8), no main effects were observed, but we continued to observe an interaction between explicitness and distance, in the same direction (β = 0.08, *t* = 2.1, *p* < 0.05). No other significant main or interaction effects were observed in the regions of analysis.

#### Discussion

The standard theory requires different mechanisms for resolving local and remote control (binding vs. contextual interpretation) and the pragmatic theory proposes the same mechanism (contextual interpretation). Hence, the standard theory predicts differences in the processing of local and remote control, while the pragmatic theory does not. As noted above, these differences might take several forms.

First, Mauner et al. (1995) suggest that syntactic resolution of PRO should have the same processing cost whether the antecedent is explicit or implicit, but that pragmatic resolution of PRO should require costly inference when the antecedent is implicit. According to these assumptions, if local control reflects a syntactically-mediated dependency and remote control reflects a pragmatically-mediated dependency, an interaction between distance and explicitness should be observed such that explicitness has an effect on processing in remote control but not in local control. In Experiment 2 we observed a significant interaction between distance and explicitness at the reason clause, but in the opposite direction: the implicit condition appeared to be costly in the *local* cases and not the remote cases. This pattern is not predicted by either the standard theory or the pragmatic theory, and it also differs from Mauner et al.'s earlier results in which no cost of explicitness was observed for local control of reason clauses.

We hypothesize that the slowdown in the implicit local condition may not reflect the cost of implicitness *per se*, but may rather have been due to the time course of processes elicited by the current materials. We assume that constructing the syntactic and thematic representation associated with the passive may take time (Chow et al., 2015). If this process is not complete by the time the reason clause is encountered, which may have been the case in the local conditions, resolution of PRO will not be immediately possible, causing temporary processing difficulty. However, in the remote conditions, the extra intervening material (*The reason was*) may have acted as a “buffer,” providing enough time for the passive sentence to be fully processed by the time the reason clause was encountered. Experiments 3 and 4 include such a buffer in both local and remote conditions and show that this eliminates the cost of implicit control in the local conditions.

Second, the standard theory assumes that local and remote control are mediated by different mechanisms (contextual interpretation and syntactic binding, respectively), and this difference in representational encoding could be reflected online in behavioral measures such as reading time as differences between local and remote configurations that are independent of explicitness—in other words, a main effect of distance.

In Experiment 2, we observed a significant main effect of distance at the infinitival and the verb in the reason clause, with faster reading times in remote conditions. That is, readers appear to be faster to process a reason clause that is syntactically independent of its target clause as compared to a reason clause whose target clause is a syntactic co-dependent within the same sentence. We refer to this as *the remote speed-up effect* of Experiment 2. These results are thus consistent with the predictions of the standard theory: contextual interpretation of PRO in a reason clause is less costly than syntactic binding.

However, there are also several alternative explanations of the remote speed-up effect, which we explore in Experiments 3 and 4. First, in remote conditions the presence of *the reason was* provided readers with extra time to process the target clause. If target clause was not fully interpreted when the reason clause appeared, processing difficulty would naturally ensue. Second, this phrase also provided readers with a cue that an infinitival reason clause may be on its way. This might facilitate resolution of PRO, and lead to faster reading times in the remote conditions. Experiment 3 tested the first possibility by adding a temporal modifier to the target clause in local conditions to better match the time course with the remote conditions. Experiment 4 tested the second possibility by adding a cue to the reason clause in the local condition (*just in order*) to parallel *the reason was* in the remote conditions.

### Experiment 3

Experiment 3 used the same design as Experiment 2 but added an intervening temporal modifier to the local conditions in order to match the distance between the target clause and the reason clause across local and remote conditions. If the overall slowdown for local conditions and the cost of implicitness for local conditions in Experiment 2 were because the target clause had not been fully processed by the time the reason clause was encountered, these differences should be eliminated in Experiment 3.

#### Methods and materials

##### Participants

Participants were 39 native speakers of English from the University of Maryland community, none of whom had taken part in the previous experiments. They either received credit in an introductory linguistics course, or were compensated $10. All participants were naïve to the purpose of the experiment. The self-paced reading task lasted for approximately 30 min.

##### Materials

Twenty-four sets of four target sentences again varied in a 2 × 2 design with the factors *explicitness* and *distance*. However, to match the amount of time available for processing between the verbs in the local and remote conditions, buffer material was included in the local target clauses, usually a temporal modifier like 3 weeks ago, as shown in Table 5. The same fillers and comprehension questions were used as in the earlier experiments. An example set of materials is provided in Table 5.

**Table 5:** **Experiment 3 materials**.

**Regions of the analysis**											
	**Pre-target**	**1**	**2**	**3**	**4**	**5**	**6**	**7**	**8**	**9**	**Post-target**
ex. loc:	The committee interviewed	the	candidates	three	weeks	ago	to	find	the	best	person for the job.
im. loc:	The candidates	were	interviewed	three	weeks	ago	to	find	the	best	person for the job.
ex. rem:	The committee interviewed	the	candidates.	The	reason	was	to	find	the	best	person for the job.
im. rem:	The candidates	were	interviewed.	The	reason	was	to	find	the	best	person for the job.

##### Procedure

The procedure for Experiment 3 was the same as the procedure for Experiments 1 and 2.

##### Data analysis

The minimum comprehension question accuracy required for inclusion of a participant's data in the analysis was 80%. Data from three participants were excluded due to comprehension question inaccuracy, resulting in a final dataset of 36 participants.

Statistical analysis was the same as described above for Experiment 2, except that because of the additional material in the local conditions, all nine regions of interest could now be analyzed with the full model including both distance and explicitness. Reading times above 2000 ms were again excluded, resulting in a loss of 0.23% of the total data.

#### Results

Mean comprehension question accuracy for experimental stimuli across participants and items was 91% (range across conditions: 89–92%), suggesting that participants were successful in comprehending the main experimental stimuli.

**Figure 3:**
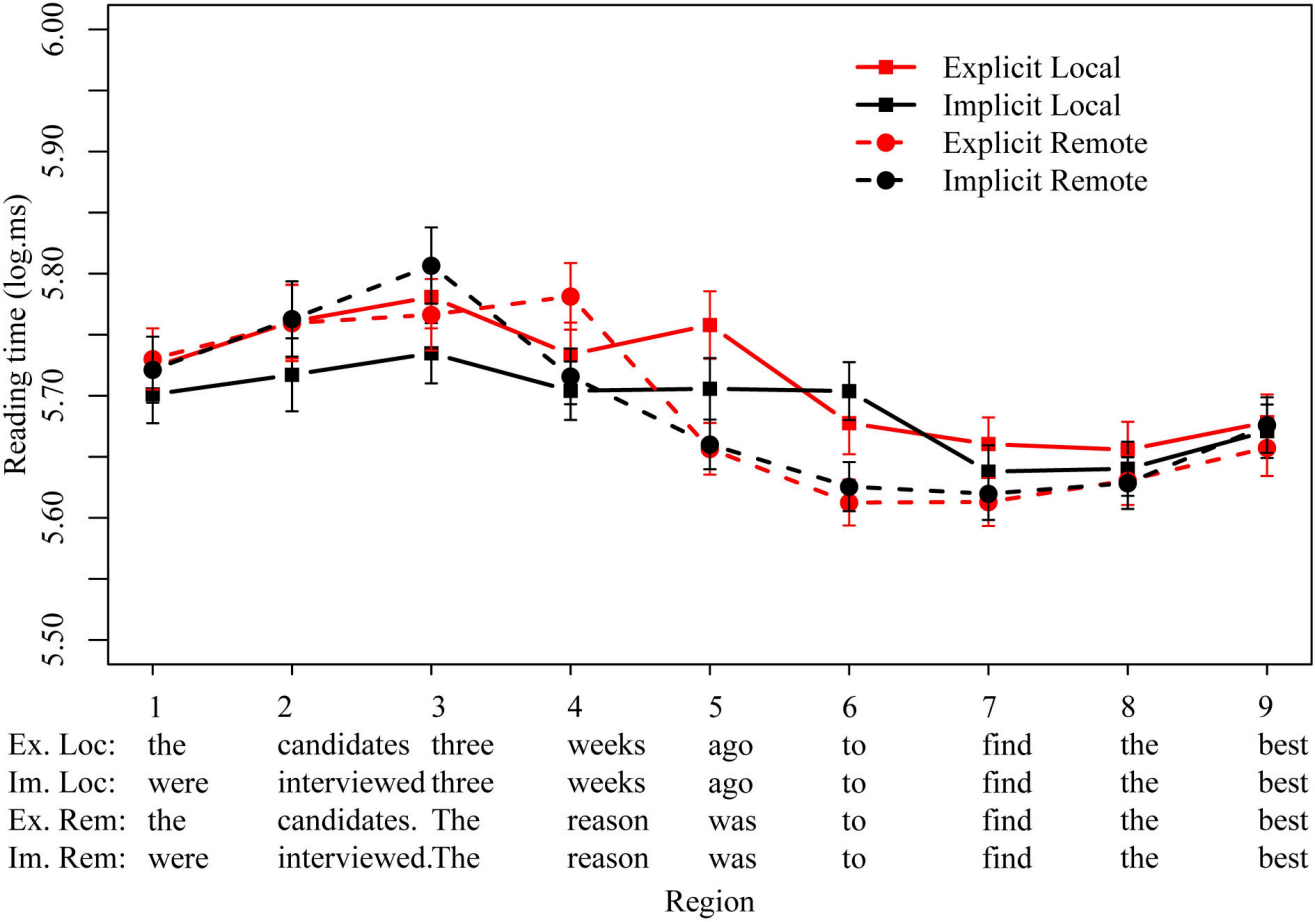
**Mean logged reading times (ms) for all four conditions in regions of interest in Experiment 3**.

Logged reading times are plotted in Figure 3, with significant effects summarized in Table 6. The reason clause began in region 6, and we observed no significant main or interaction effects in preceding regions 1–4. However, region 5, just prior to the infinitival (*was* in the remote conditions and the last word of the temporal modifier in the remote conditions) showed a strong main effect of distance (β = 0.1, *t* = 3.7, *p* < 0.001) due to faster reading times in remote conditions, and a marginal main effect of explicitness (β = 0.05, *t* = 1.9, *p* = 0.06) that appeared to be due to slower reading times in the explicit local condition.

**Table 6:** **Experiment 3 results**.

**Region**	**Factors**	**Estimate**	**S.E**.	***t*-value**	***p*-value**
	Explicitness	−0.054	0.028	−1.9	0.058
5	Distance	−0.104	0.028	−3.67	0.0003
ago/was	Explicitness × Distance	0.057	0.04	1.44	0.151
	Explicitness	0.026	0.026	1	0.318
6	Distance	0.065	0.026	−2.5	0.013
*to*	Explicitness × Distance	−0.013	0.037	−0.35	0.724
	Explicitness	−0.023	0.025	−0.95	0.344
7	Distance	−0.048	0.025	−1.95	0.051
*find*	Explicitness × Distance	0.03	0.035	0.86	0.388

At the infinitival *to* and the subsequent verb (region 6–7), we see no sign of any main effects or interactions involving explicitness, but we again observe faster reading times in the remote conditions (region 6: β = 0.07, *t* = 2.5, *p* < 0.05, region 7: β = 0.05, *t* = 2.0, *p* = 0.05). Importantly, we observe no effect of implicitness within the local conditions, in contrast with Experiment 2. That is, in both local and remote conditions, readers are just as fast to process reason clauses whether they follow a short passive or an active target clause.

#### Discussion

Experiment 3 was designed to better understand two differences between local and remote conditions that were observed in Experiment 2. First, Experiment 2 showed an interaction between distance and explicitness at the reason clause, such that the local conditions showed a cost of implicitness but the remote conditions did not. Because this pattern was predicted by neither the standard theory nor the pragmatic theory, we suspected that it reflected the fact that the passive had not been fully processed by the time the reason clause appeared in the local condition. The results of Experiment 3 are consistent with this hypothesis, as when a temporal modifier was added as a “buffer” between the target clause and the reason clause, no cost of implicitness was observed at the reason clause in the local conditions. This result is thus in keeping with earlier findings concerning local control (Mauner et al., 1995).

Importantly, neither Experiment 2 nor Experiment 3 showed any evidence of one possible pattern of processing differences between local and remote control that might have been predicted by the standard theory. If, as suggested by Mauner et al. (1995), resolving control when the antecedent is implicit requires costly inference when control is pragmatically mediated but not when it is syntactically mediated, then the standard theory would predict a cost of implicitness for remote control and not for local control. This prediction is not borne out by the current results, which show no sign of processing cost for implicitness in the remote control conditions.

In Experiment 2 we also observed overall faster reading times at the reason clause in remote control compared to local control. This pattern would also be consistent with the standard theory if the processes involved in resolving the pragmatic dependency are faster or less effortful than the processes involved in resolving the syntactic dependency. However, this pattern could also have simply been due to the fact that the extra intervening material between the target and reason clause in the remote conditions (*the reason was*) might have provided more time to fully process the target clause.

The results of Experiment 3 appear to argue against this alternative explanation, because when we control for timing between the local and remote conditions, we continue to observe a strong effect of distance in the reason clause, once again with faster reading times in remote as compared to local reason clauses. However, we also noted another alternative explanation for the facilitated processing of remote control observed here, which is that the content of the intervening material in *the reason was* provided a predictive semantic cue for the upcoming infinitival reason clause. The temporal modifier included in the local conditions in Experiment 3 provided additional processing time, but did not include this kind of semantic cue. In support of this explanation, in Experiment 3 significantly longer reading times were also observed for local relative to remote conditions in the region immediately prior to the reason clause. This early effect cannot be driven by control *per se*, but could be explained if the predictability of *the reason was* sped up reaction times in the remote condition relative to the less predictable temporal modifier (e.g., 3 weeks ago) in the local condition. Experiment 4 was designed to address this remaining discrepancy by making the material immediately preceding the reason clause equally predictable across conditions.

### Experiment 4

Experiment 4 used the same design as Experiments 2 and 3 but used the phrase *just in order to* in the local conditions such that both local and remote conditions contained a semantic cue that could be used to predict or prepare for the upcoming reason clause. If the faster reading times in the remote conditions observed in Experiments 2 and 3 were due to the presence of the semantic cue *The reason was*, this difference in processing time should be eliminated in Experiment 4.

#### Methods

##### Participants

Participants were 38 native speakers of English from the University of Maryland community, none of whom had taken part in the previous experiments. They received credit in an introductory linguistics course for participating. All participants were naïve to the purpose of the experiment until after participating, when an explanation was provided. The self-paced reading task lasted for approximately 30 min. The task was performed as part of a 1 h session involving an unrelated experiment.

##### Materials

Twenty-four sets of four target sentences again varied in a 2 × 2 design with the factors *explicitness* and *distance*. However, we included *just in order* in the local conditions in Experiment 4 to match not only the time course, but also the predictiveness of the upcoming reason clause in remote and local conditions. The same fillers and comprehension questions were used as in the earlier experiments. An example set of materials is provided in Table 7.

**Table 7:** **Experiment 4 materials**.

**Regions of the analysis**											
	**Pre-target**	**1**	**2**	**3**	**4**	**5**	**6**	**7**	**8**	**9**	**Post-target**
ex. loc:	The committee interviewed	the	candidates	just	in	order	to	find	the	best	person for the job.
im. loc:	The candidates	were	interviewed	just	in	order	to	find	the	best	person for the job.
ex. rem:	The committee interviewed	the	candidates.	The	reason	was	to	find	the	best	person for the job.
im. rem:	The candidates	were	interviewed.	The	reason	was	to	find	the	best	person for the job.

##### Procedure

The procedure for Experiment 4 was the same as that described above.

##### Data analysis

The minimum comprehension question accuracy required for inclusion of a participant's data in the analysis was 80%. Data from one participant were excluded due to comprehension question inaccuracy. Data from one participant who was not a native speaker of English were also excluded.

Statistical analysis was the same as described above for Experiment 3. Reading times above 2000 ms were excluded, resulting in a loss of 0.28% of the total data.

#### Results

Mean comprehension question accuracy for experimental stimuli across participants and items was 93% (range 92–95%), suggesting that participants were successful in comprehending the main experimental stimuli.

**Figure 4:**
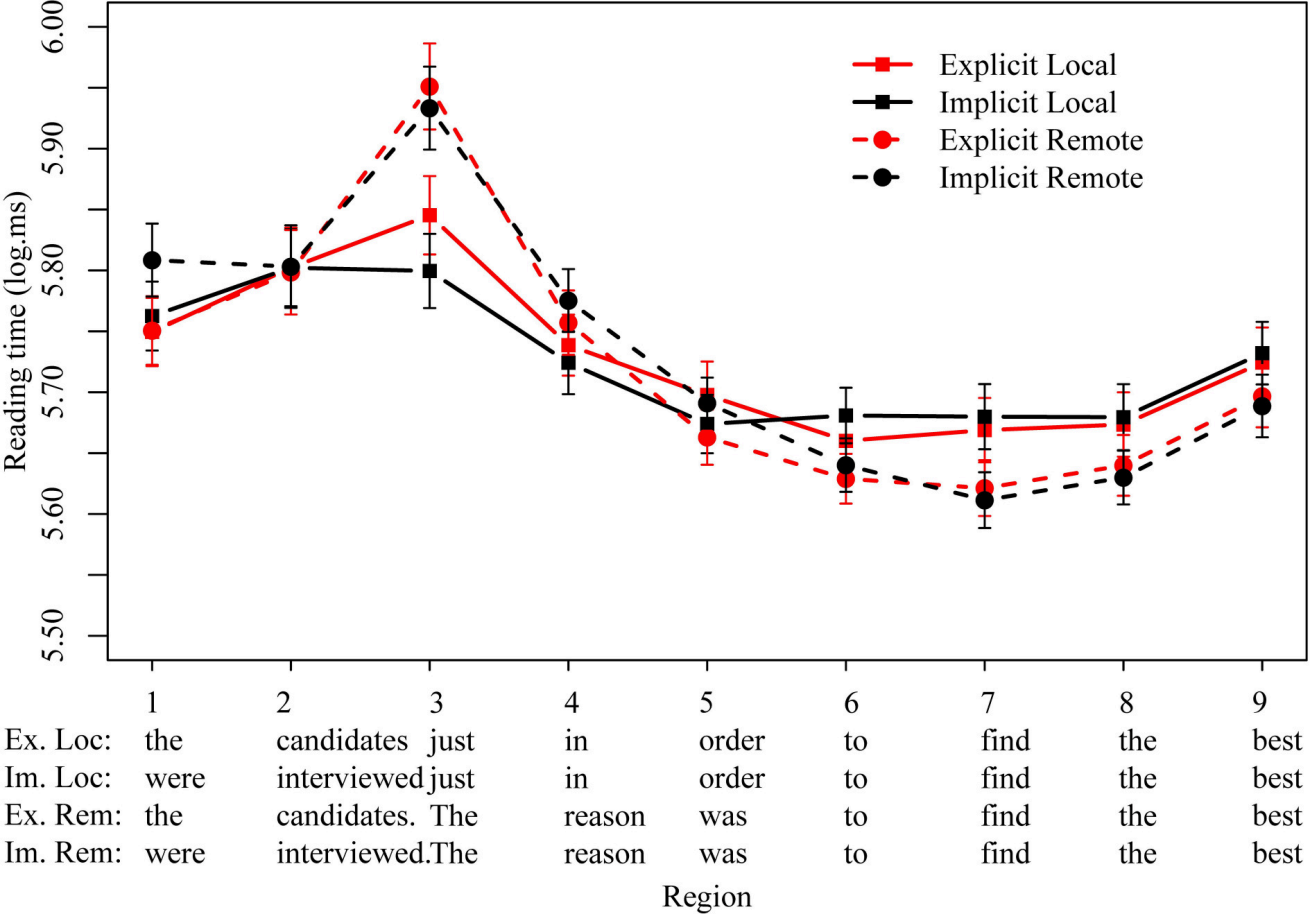
**Mean logged reading times (ms) for all four conditions in regions of interest in Experiment 4**.

Logged reading times are plotted in Figure 4, with significant effects summarized in Table 8. In the regions preceding the reason clause (1–5) we observed no significant differences except for a main effect of distance due to slower reading times in the remote conditions in region 3 (β = 0.1, *t* = 3.1, *p* < 0.01), which corresponded to the sentence-initial determiner *the* in remote conditions and *just* in the same region in local conditions. This difference may be associated with the presence of the sentence boundary in the remote conditions.

**Table 8:** **Experiment 4 results**.

**Region**	**Factors**	**Estimate**	**S.E**.	***t*-value**	***p*-value**
	Explicitness	−0.053	0.034	−1.57	0.117
3	Distance	0.105	0.034	3.12	0.002
just/The	Explicitness × Distance	0.027	0.048	0.58	0.56
	Explicitness	0.011	0.026	0.42	0.674
7	Distance	−0.048	0.026	−1.81	0.07
find	Explicitness × Distance	−0.021	0.037	−0.56	0.573

No other regions showed a significant effect of distance. In particular, at the infinitival *to* and the verb in the reason clause (regions 6–7), we again observed no sign of any main effects or interactions between distance and implicitness. Reading times were numerically faster in remote vs. local conditions in regions 6–9 (*to find the best*). However, in Experiment 4, these differences were small and unreliable, eliciting only a marginal effect of distance in region 7 (β = 0.04, *t* = 1.8, *p* ≤ 0.07; all other regions *p* = 0.2)^7^.

#### Discussion

Experiment 4 examined whether processing differences would be observed in the resolution of reason clauses in local and remote control configurations when both local and remote conditions included a semantic cue for the upcoming reason clause. As the remote conditions necessarily include such a cue in the phrase *the reason was*, in Experiment 4 we included the phrase *just in order* in the local conditions to balance both the distance between the target and the reason clause as well as the presence of a semantic cue for the upcoming reason clause across conditions. Controlling for both timing and predictiveness in this way, we observe no reliable differences in reading times in the reason clause as an effect of whether its target is local or remote, although we continue to observe a trend in this direction. This suggests that much of the “remote speed-up” effect that was observed in Experiments 2 and 3 was due to differences in the extent to which the upcoming reason clause was cued by the prior context, rather than differences between the local and remote configurations in the difficulty of resolving the reason clause.

Because the standard theory proposes different mechanisms for resolving local and remote control (binding vs. contextual interpretation), it predicts differences in the processing of these two configurations; but the pragmatic theory, which proposes the same mechanism (contextual interpretation), does not. In Experiment 4 we observe no such differences in the effect of explicitness on processing of remote vs. local control as reflected in reading times, and no effect of distance in the reason clause. Therefore, to the extent that differences in representation should be reflected as differences in constructing such a representation in online processing, the absence of such differences in Experiment 4 raise a potential challenge for the standard theorist. As we discuss in more detail below, several responses are possible on behalf of the standard theory; it could be that reading times in particular are not a sensitive enough measure to detect differences between local and remote control, or that processing cost more generally does not index whether a dependency is semantically or pragmatically mediated. To the extent that either of these responses are adopted, however, they undermine some earlier arguments in favor of the standard theory.

## General discussion

In four self-paced reading time experiments, we examined the processing of infinitival reason clauses, in contexts that favor anaphoric construals of PRO, their understood subject. In our materials, the likely referent for PRO is always the individual who satisfies the deep-S role for the preceding target clause; for example the interviewer when the target clause has *interview* as its verb. We compared explicit control, where this role is linked to an audible noun phrase, to implicit control, where it is not. In implicit conditions, the target clause is a short passive. Already Mauner et al. (1995) made this comparison in the local configuration, where the reason and target clauses are syntactically dependent. Ours is the first study to do this for the remote configuration as well, where the two are in separate sentences. What we found, in summary, is this. First, reading times at the reason clause were not longer when the antecedent was implicit relative to when it was explicit, once we controlled the length and content of what intervenes between target and reason clauses across conditions, as in Experiment 4. For local configurations, this agrees with the findings of Mauner and colleagues. That study too found no significant differences in relevant regions between implicit and explicit control, on measures that did distinguish both from cases where, offline, control is judged unacceptable. Our new finding is that no such differences are observed in the inter-sentential remote configuration either, where one might have thought that a costly pragmatic inferencing operation would be required. Second, we also did not observe significant main effects of distance between local and remote control when both the length and the content of intervening material were matched.

Our results bear on a question of grammatical representation. When the understood referent of PRO in a reason clause is an individual mentioned or implied by the target clause, what sort of relation does PRO have to that clause? Is it syntactically linked to an argument there? Or is this a kind of discourse anaphora, with PRO here ranging over a specially restricted domain? On the standard theory (Roeper, 1987), the same relation underlies both explicit and implicit control. This much is consistent with our results, and with those in Mauner et al. (1995), none of which show any relevant effects of the difference. However, the standard theory also takes the common relation to be syntactic, a binding relation between PRO and an argument in the target clause. Such a syntactic link is possible in the local configuration, since the reason clause is adjoined to the target clause. But it is not possible in the remote configuration, since the two clauses are independent. Therefore, if reason control is syntactic when local, as the standard theory says, it must have a different analysis when remote; and if it has the same analysis either way, it cannot be syntactic, and must in both cases be mediated by discourse. Thus, given the standard theory of reason control, we expect a main effect of distance, local vs. remote, on some online measure, while on a uniformly pragmatic theory we do not. On our reading-time measure we found no such effect, not once we controlled for both timing and predictiveness across conditions, as in our Experiment 4. Thus, our results fail to confirm the standard theory.

More importantly, the current results subvert the earlier argument for the standard theory from processing measures. In past work, both the self-paced-reading-time and the stop-making-sense task showed no relevant difference between implicit and explicit control in local configurations (Mauner et al., 1995), while processing costs were observed in baseline conditions (intransitives and middles) in which control of reason clauses appears unacceptable. These data were taken as evidence that both implicit and explicit control were syntactic dependencies. We agree that a similar processing profile may suggest that these are dependencies of the same sort. But the current work illustrates that these prior data cannot be taken to argue that both are syntactic dependencies, since remote control cannot be syntactic, and there too our measures do not distinguish implicit from explicit control.

While these results thus remove a previous argument in favor of the standard theory, they challenge the standard theory directly only if we think that self-paced reading times are sensitive to the difference between syntactic vs. pragmatic anaphora. But as discussed in Section Background, they may not be. Indeed, perhaps these two routes to interpretation are not reliably distinguished by processing cost (see Cunnings et al., 2014, for discussion), or any existing measure of processing. In the latter case there could be no processing evidence for the analysis of control. However, either observation weakens not only our own conclusions, but also the earlier defense of the standard theory. That defense was primarily based on behavioral processing measures (stop-making-sense task and reading times) that were not independently demonstrated to distinguish binding from free anaphora. Hence, our results either provide direct evidence against the standard theory, or undermine earlier arguments in its favor, depending on the evidentiary status of reading times.

Our experiments also highlight the importance of several design factors. Experiment 1 suggested that narrow focus in target clause can increase reading times in the reason clause, a construction whose semantics is sensitive to focus (Dretske, 1972). We believe this makes the long-passive a poor baseline for comparison with implicit control, since normally the *by*-phrase carries narrow focus. Experiments 2 and 3 illustrated the importance of controlling both the length and content of what comes between the target and reason clauses. Reading times for the infinitival verb in the reason clause are slower when it immediately follows the target clause than when it is separated from the target clause by a buffer, either a temporal adjunct in local configurations, or *the reason was* in remote configurations. This may reflect the time it takes to process the passive target clause (Chow et al., 2015). Even with a temporal buffer, reading times were still faster at the infinitive when the buffer is predictive of a reason clause (*the reason was*) than when it is not (*3 days ago*). Yet reading times at the infinitive did not differ significantly between remote and local control in Experiment 4, where we matched the buffers for both length and content, pairing *the reason was* in remote conditions with *just in order* in local conditions.

To finish, let us turn briefly to the issue of implicit arguments. Our results undermine earlier arguments in favor of the standard theory. Although they do not prove an alternative pragmatic theory correct, they suggest that further investigation of this kind of account would be worthwhile. As we note in the introduction, in a pragmatic theory many of the constraints on control of reason clauses could be captured by a domain condition that is not syntactic but conceptual. Anaphoric uses of PRO in a reason clause denote the individual(s) viewed as responsible for the fact that the reason clause is meant to explain (Farkas, 1988; Landau, 2000; Williams, 2015). This condition is manifest in cases like (10), where PRO finds its antecedent in the surface subject of a passive: the referent of PRO must be viewed as responsible for what happened (Williams, 1974; Zubizaretta, 1982; Roeper, 1987). To be adequate, such a theory would need to say, for example, that the referent of a direct object is never viewed as responsible for the fact expressed by its clause. If it does, a silent argument in passives would play no role in explaining implicit control. Having weakened some of the previous motivation for the standard theory, we suggest that future research ought to explore such pragmatic alternatives in greater detail.

## Author contributions

AW and MM designed the experiments. MM and JG implemented the experiments. JG and EL analyzed the data. AW, EL, and MM wrote the paper. All four authors participated in editing and revising the manuscript.

### Conflict of interest statement

The authors declare that the research was conducted in the absence of any commercial or financial relationships that could be construed as a potential conflict of interest.

## Footnotes

^1^Binding of PRO is normally called *control*. But we use “control” more neutrally, just to denote the resolution of PRO's reference, whether or not this is decided by binding.

^2^Control by objects is possible, however, for infinitival “purpose clauses” (Faraci, 1974; Williams, 1974; Bach, 1982; Jones, 1985). An example is *Maria brought Mary along to translate*, where the translator is Mary, not Maria. Jones (1985) distinguishes these from reason (rationale) clauses in three further ways, following Williams (1974) and Faraci (1974). Only reason clauses have or permit *in order to*. Only reason clauses can be preposed to sentence-initial position. And only purpose clauses can have a gap in their VP, bound by an argument in the main clause, as in *Mary brought a pen to write with*, where *a pen* binds a gap after *with*. But see Whelpton (2002) for concerns about this taxonomy.

^3^There is a strong case for silent arguments with an anaphoric or “definite” (Fillmore, 1986) interpretation (Partee, 1989; Condoravdi and Gawron, 1996). But the silent argument in a short passive would not be anaphoric or definite. Its interpretation would be equivalent to a narrow-scope existential quantifier: “The candidates were interviewed carefully by *someone*.” And the case for such arguments is much weaker (Williams, 2015).

^4^For example, the subject of the copular clause might contain a silenced genitive pronoun, [*his*] *reason*, or a silent relative clause elided under identity with the target clause, *the reason* [*that the candidates were interviewed*]. Then the clause would to be restructured at an unpronounced level of syntax, so that the silent binder is in a local relation to PRO. On the special grammar of copular clauses, see the review in Mikkelsen (2011).

^5^A referential dependency that is intersentential cannot be syntactic, but it may still be sensitive to structural properties of the antecedent. VP Ellipsis is sensitive to voice, for example, and pronominal reference may be sensitive to gender class. Neither of these cases is itself a good model for remote control. But it remains coherent to claim both that PRO in a reason clause is a free anaphor, and that its resolution is sensitive to something like subjecthood. We return to this in passing in the General Discussion.

^6^Constructional restrictions on the domain of a pronoun are not in the standard semantic toolkit, and therefore much more would need to be said. For thoughts see Landau (2000) and Williams (2015), which refine a suggestion in Farkas (1988), with roots in Williams (1974). Also see Whelpton (2002) in opposition, and the discussion in Sag and Pollard (1991).

^7^In Experiment 3, we observed a slow-down in reading times at the first word in the second sentence (“The”) in remote conditions, while the same effect did not obtain in Experiment 4. Hence, as a reviewer pointed out, it is precisely when we do not see sentence-boundary effects that we find no advantage for remote control, as in Experiment 4. Whether this is due to a relationship between sentence-boundary effects and implicit control, or some incidental difference between the two experiments, we leave for future investigation.
